# Association between sleep duration and obesity is age- and gender-dependent in Chinese urban children aged 6–18 years: a cross-sectional study

**DOI:** 10.1186/s12889-015-2359-0

**Published:** 2015-10-07

**Authors:** Muqing Cao, Yanna Zhu, Baoting He, Wenhan Yang, Yajun Chen, Jun Ma, Jin Jing

**Affiliations:** Department of Maternal and Child Health, Faculty of Public Health, Sun Yat-sen University, No. 74, Zhongshan 2nd Road, Yuexiu, 510080 Guangzhou, People’s Republic of China; Institute of Child and Adolescent Health, School of Public Health, Peking University Health Sciences Center, Beijing, People’s Republic of China

**Keywords:** Sleep, Obesity, Gender, Age, Children

## Abstract

**Background:**

Information on the relationship between sleep duration and obesity among children in urban Guangzhou, China is limited. This study aims to examine the relationship between sleep duration and obesity in children aged 6–18 years.

**Methods:**

The sample consisted of 11,830 children aged 6–18 years. The children were randomly selected from 13 schools in three urban districts of Guangzhou. The study was conducted from September to November 2013. The height and weight of the children were measured. Adiposity status was estimated using body mass index and according to the cut point in China criteria. In the structured questionnaire, children reported daily sleep hours (less than 7 h, 7–9 h and more than 9 h), weekly food intake amount (including vegetables, fruit, sugar beverages and meat), physical activity and sedentary time. A caretaker would answer the questionnaire on behalf of a child aged below nine.

**Results:**

A total of 8,760 children (49.0 % boys) completed the study. The prevalence of obesity was 8.4 % (9.8 % in boys and 5.7 % in girls). Adjusted for age, diet and physical activity/sedentary behaviour, the odds ratio (OR) for obesity comparing sleeping <7 h (short sleep duration, SSD) with ≥9 h (long sleep duration, LSD) was 0.70 (95 % CI: 0.69–0.72) among boys and 1.73 (95 % CI: 1.71–1.74) among girls. Stratified by age, OR for boys aged 6–12 years comparing SSD with LSD was 0.60 (95 % CI: 0.55–0.66); by contrast, OR was 1.33 (95 % CI: 1.30–1.37) for boys aged 13–18 years.

**Conclusion:**

Short sleep duration is associated with increased chances of obesity among girls and 13- to 18-year-old boys, but the chances of obesity are decreased among 6- to 12-year-old boys. Age and gender should be regarded as specific characteristics for the effects of short sleep on obesity.

## Background

Sleep is an important modulator of growth, maturation and health of children and adolescents [1]. However, as a hallmark of modern society, sleep duration is decreasing for both children and adults in the last few decades [2]. In the 1960s, the average sleep duration in the United States was more than 8 h [3], but dropped to 7 h in 1995 [4]. In 2005, a survey conducted on children showed that the average sleep duration lasted from 8.4 h (11–12 years old) to less than 7 h (16–17 years old) in the United States [5]. Reports from China, Australia and other countries [6, 7] also indicated a decreasing sleep duration among children in recent years. Short sleep duration is indeed a global phenomenon.

Short sleep duration is associated with neuroendocrine and metabolic modification, including decreased levels of leptin, glucose tolerance and insulin sensitivity, as well as increased levels of ghrelin, hunger and appetite [8]. Moreover, evidence suggests the linkage between short sleep and specific behaviour, such as low physical activity and low consumption of fruit and vegetables [9]. Metabolic change and behaviour are strongly associated with obesity, and thus raise the concern if short sleep duration is a risk factor of childhood obesity.

Many studies have explored the relationship between sleep duration and obesity. Several cross-sectional studies have reported that short sleep duration is associated with childhood obesity [6, 10–13]. With regard to prospective studies, a meta-analysis that reviewed 22 longitudinal studies revealed twice the risk of obesity in subjects reporting short sleep [14]. However, the age ranges within the published studies typically vary from preschool children to adolescents. Sleep duration changes as a child grows to adulthood and generally decreases when children get older [6]. However, reports using objective measurement indicated that sleep duration increases from mid- to late adolescence [15]. In this case, age should be considered when an epidemiologic study is conducted. Gender is another factor that should be taken into account. The requirements for daily sleep duration may also vary due to the different hormone levels of boys and girls. In addition, gender-specific factors that can influence the prevalence of obesity remain unknown. Moreover, obesity is not only affected by sleep, but is also modulated by physical activity, diet, habit and other behavioural factors that are difficult to quantify [16].

Although the relationship between sleep duration and obesity has been extensively investigated in children, information on the age- and gender-specific effect is limited. Additionally, this association remains obscure in the Chinese children population. Given this background, we explored whether the relationship between sleep duration and obesity is age- and gender-dependent in the Chinese children. We conducted a cross-sectional study among children aged 6–18 years from September to November 2013 in the urban area of Guangzhou. Our study investigated (i) the up-to-date prevalence of obesity, (ii) distribution of sleep duration and (iii) association between obesity and short sleep duration in different age and gender groups. For possible confounding factors, we adjusted physical activity/sedentary behaviour and dietary intake.

## Methods

### Sampling and data sources overview

Data were obtained from the study of Student Constitution and Health, which was conducted in 2013 in Guangzhou, a city in south China. Multistage cluster sampling was used. Ten districts in Guangzhou were randomly selected by computer, and the random sequence of schools was computer-generated and stratified by (1) school district, (2) school grades (primary, middle, or high) and n school size in each district. All of the students in the selected schools in grades 1 to 12 were invited to participate in the survey. After a school is confirmed to be eligible, informed consent was obtained from parents and children. Thirteen schools, which included 11,830 children, agreed to participate in the study. The Ethical Committee of School of Public Health, Sun Yat-sen University approved this study.

### Anthropometric measurement

Each child underwent an anthropometric measurement from September to November 2013. A qualified technician measured the children’s height (centimetre, cm) and weight (kilogram, kg). All of the measurements were conducted in the school where the children studied. A metal column height measuring stand (precision: 0.1 cm) was used in measuring heights. Children were required to stand erect with back, buttocks and heels in continuous contact with the vertical height rod of the stadiometer and head orientated in the Frankfurt plane. The horizontal headpiece was then placed on top of the child’s head to measure the height. When weight was measured, children were required to wear underwear and to stand on the lever scale (precision: 0.1 kg) at ease. The height and weight of each child were measured twice, and the average numbers were recorded to decrease error. Data of children suffering from obvious diseases or physical/mental deformities were excluded.

### Questionnaire survey

A self-reported questionnaire was designed to obtain information on gender, date of birth, date of survey, daily food intake (including fruit, vegetables, sugar beverages and meat) daily physical activity/sedentary behaviour and daily sleep duration. From September to November 2013, inspectors who were familiar with the questionnaire went to schools and distributed the questionnaire. Children enrolled in the study were gathered and answered the questionnaire under supervision of the inspectors. Any question or confusion from children was clarified to ensure that every child understood all of the items. The completed questionnaires were checked for quality control. If the child was under nine years old, one of the parents would answer the questionnaire on the child’s behalf to prevent inaccurate information. Questionnaires were distributed in class, and children were instructed to give the form to their parents. Parents were provided with instructions on answering the questionnaire via telephone or message. The team ensured that children would bring back the answered questionnaire to school. When all of the questionnaires were handed in, researchers would collect them from each class, and quality control would be performed. We emphasized that the first caregiver of the child should answer the questionnaire for he or she knew the child well. If the caregivers had any questions, then they should check with the child. Questionnaire items are described as follows.

*Sleep duration* was assessed by the question, ‘How many hours each day do you spend sleeping?’ Sleep duration was categorized as <7 h, 7–9 h and ≥9 h.

### Daily food intake

Fruit intake was assessed by the question, ‘How many servings of fruit do you usually eat each day? One serving of fruit has the size of an adult’s fist’.

Vegetable intake was assessed by the question, ‘How many servings of vegetables do you usually eat each day? One serving of vegetable has the size of an adult’s fist’.

Sugar beverage consumption was assessed by the question, ‘How many servings of sugar beverages do you usually take each day? One serving of sugar beverage is 250 millilitres’. Sugar beverage includes soda (i.e. Coca-Cola), energy drinks (i.e. Red Bull), milk drinks, juice with sugar and other beverages that contain sugar.

Meat intake was assessed by the question, ‘How many servings of meat do you usually eat each day? One serving of meat has the size of an adult’s palm’.

### Daily physical activity and sedentary behaviour

High level physical activity (HLPA) was assessed by the question, ‘How many hours each day do you usually spend in high level physical activity? High level physical activity means activity that causes people to be out of breath, perspire and experience extreme exhaustion, such as basketball, football, carrying a heavy load etc.’.

Middle level physical activity (MLPA) was assessed by the question, ‘How many hours each day do you usually spend in middle level physical activity? Middle level physical activity means activity that causes people to mildly perspire and experience slight exhaustion, such as bicycling, playing table tennis, badmintonetc. but not walking’.

Walking was assessed by the question, ‘How many hours each day do you usually spend in walking? Walking includes those that happened at school, home, commute between school and home, and for exercise’.

Sedentary behaviour was assessed by the question, ‘How many hours each day do you usually spend in sitting or lying still at school and home (excluding sleeping)?’

The questionnaire has not been statistically validated nor tested for reliability.

### Definitions of obesity

Body mass index (BMI) was calculated by dividing weight in kilograms by height in metres squared (kg/m^2^). Obesity was defined according to the latest Chinese criteria (Table 1) [17].

**Table 1:** The cut-off points for obesity (BMI, kg/m^2^) in children aged 6–18 years old in China [17]

Age (years)	Boys	Girls
6.0	18.4	18.4
6.5	18.8	18.6
7.0	19.2	18.8
7.5	19.6	19.1
8.0	20.1	19.5
8.5	20.6	19.9
9.0	21.1	20.4
9.5	21.7	20.9
10.0	22.2	21.5
10.5	22.7	22.1
11.0	23.2	22.7
11.5	23.7	23.3
12.0	24.2	23.9
12.5	24.6	24.4
13.0	25.1	25.0
13.5	25.5	25.5
14.0	25.8	25.9
14.5	26.2	26.3
15.0	26.5	26.7
15.5	26.8	27.0
16.0	27.0	27.2
16.5	27.3	27.4
17.0	27.5	27.6
17.5	27.8	27.8
18.0	28.0	28.0

### Statistical analysis

Data were inputted by Epidata 3.0 software (The EpiData Association, Odense, Denmark) and analyzed by Statistical Package for the Social Sciences (SPSS, version 13, IBM Corporation, New York). Descriptive statistics were calculated for all of the variables, including continuous variables (presented as mean values and standard errors) and categorical variables (presented as proportions). The differences of categorical variables were evaluated by chi-square tests. For continuous variables, t- tests were used to evaluate gender differences. One-way ANOVA was employed to evaluate differences among sleep duration groups, and post hoc comparison between groups was performed via Student–Newman–Keuls tests. The association between sleep duration and risk of obesity was evaluated by logistic regression models, which were initially adjusted for age and gender; dietary intake, physical activity and sedentary behaviour were subsequently introduced into the model. Cluster data from 13 schools were also introduced into the model, and robust standard error was used to minimize the effect that data obtained from different schools may have. The results were reported by odds ratios (ORs) and corresponding 95 % confidence intervals (CIs). *P* values less than 0.05 were considered statistically significant.

## Results

### Baseline characteristics of the study population

**Fig. 1:**
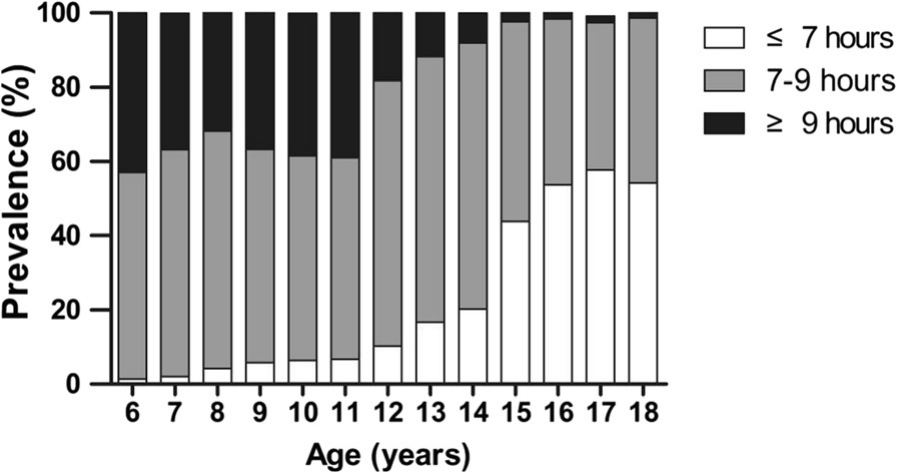
Distribution of sleep duration among children aged 6–18 years in urban area of Guangzhou, China

The sample comprised 8,760 children aged 6–18 years (mean 11.42 years, 49 % boys). The baseline characteristics of the participants, such as anthropometry, dietary intake, physical activity/sedentary behaviour, sleep duration and prevalence of obesity, are described (see Table 2). Compared with girls, boys had higher mean values of height, weight, BMI, daily intake of foods (vegetables, meat and sugar beverages) and physical activity (all *p* < 0.05, Table 2), but lower mean value of sedentary behaviour (*p* < 0.05, Table 2). The overall prevalence of obesity was 8.4 % (9.8 % in boys, 5.7 % in girls, *p* < 0.001). In total, 17.9 % of the participants reported sleep duration less than 7 h, whereas 23.1 % reported more than 9 h each day. More girls had shorter sleep duration than boys (see Table 2). The trend showed that children tend to have less sleep hours as they grow up (see Fig. 1), and gender-specific patterns are similar in boys and girls.

**Table 2:** Baseline characteristics among children aged 6–18 years in urban Guangzhou, China

	Total (*n* = 8760)	Boys (*n* = 4293)	Girls (*n* = 4467)	*P* value
Age(year)	11.42(3.39)	11.40(0.051)	11.44(0.051)	0.164
Height(cm)	146.23(19.18)	147.56(0.31)	144.91(0.27)	<0.001
6–12 years	132.12(11.76)	132.18(0.24)	132.06(0.25)	0.728
13–18 years	161.30(13.16)	164.49(0.30)	158.23(0.20)	<0.001
Weight(kg)	40.33(15.74)	42.06(0.25)	38.62(0.22)	<0.001
6–12 years	29.23(9.63)	30.27(0.20)	28.17(0.20)	<0.001
13–18 years	52.19(11.86)	55.04(0.29)	49.46(0.21)	<0.001
BMI(kg/m^2^)	18.16(4.84)	18.52(0.068)	17.80(0.078)	0.034
6–12 years	16.48(4.60)	17.00(0.078)	15.95(0.112)	<0.001
13–18 years	19.95(4.44)	20.19(0.102)	19.71(0.091)	0.01
Dietary intake (servings/day)				
Fruit	1.23(1.12)	1.22(0.018)	1.25(0.016)	0.062
6–12 years	1.23(1.104)	1.20(0.024)	1.26(0.023)	0.059
13–18 years	1.24(0.018)	1.24(0.026)	1.23(0.024)	0.790
Vegetable	1.93(1.61)	1.94(0.025)	1.92(0.024)	0.011
6–12 years	1.76(1.49)	1.76(0.031)	1.75(0.031)	0.739
13–18 years	2.11(1.71)	2.13(0.039)	2.09(0.035)	0.488
Meat	1.61(1.46)	1.77(0.024)	1.45(0.019)	<0.001
6–12 years	1.40(1.29)	1.49(0.030)	1.30(0.025)	<0.001
13–18 years	1.84(1.59)	2.08(0.039)	1.60(0.029)	<0.001
Sugar beverage	0.37(0.73)	0.46(0.013)	0.29(0.008)	<0.001
6–12 years	0.21(0.44)	0.25(0.011)	0.17(0.007)	<0.001
13–18 years	0.55(0.92)	0.70(0.024)	0.41(0.014)	<0.001
Physical Activity (PA, hour/day)				
HLPA	0.44(0.64)	0.52(0.011)	0.35(0.008)	<0.001
6–12 years	0.41(0.58)	0.45(0.013)	0.36(0.011)	<0.001
13–18 years	0.47(0.70)	0.60(0.018)	0.34(0.012)	<0.001
MLPA	0.40(0.61)	0.43(0.010)	0.37(0.008)	<0.001
6–12 years	0.39(0.58)	0.40(0.012)	0.39(0.012)	0.452
13–18 years	0.41(0.64)	0.46(0.016)	0.36(0.012)	<0.001
Walking	0.87(1.18)	0.92(0.020)	0.81(0.016)	<0.001
6–12 years	0.75(1.16)	0.77(0.025)	0.74(0.023)	0.490
13–18 years	0.98(1.19)	1.10(0.030)	0.88(0.021)	<0.001
Sedentary	4.35(3.78)	4.28(0.057)	4.43(0.058)	0.008
6–12 years	4.09(3.54)	4.06(0.073)	4.11(0.075)	0.622
13–18 years	4.63(4.00)	4.51(0.087)	4.75(0.087)	0.054
Sleep duration (%)				
<7 hours	17.9	15.4	20.2	<0.001
7–9 hours	59.0	60.7	57.5	
>9 hours	23.1	23.9	22.3	
Obesity (%)	8.4	9.8	5.7	<0.001
6–12 years	6.6	8.1	5.2	
13–18 years	10.1	13.4	6.8	

*BMI* body mass index, *PA* physical activity, *HLPA* high level physical activity, *MLPA* middle level activity

Continuous variables are displayed as mean ± standard error(boys and girls) and mean ± standard deviation(total), categorical variables are displayed as proportion

*P* values are from T-tests (continuous variables) and chi-square tests (categorical variables) between boys and girls

### Sample characteristic across sleep duration groups

Compared with children who had long sleep duration (LSD, >9 h/night), participants who had short sleep duration (SSD, <7 h/night) had increased age, height, weight and BMI (all *p* < 0.05, Table 3). For dietary intake, SSD children had a lower daily intake of fruit (*p* < 0.05, Table 3) but a higher daily intake of meat and sugar beverages (both *p* < 0.05, Table 3) compared with LSD children. For physical activity, daily high level physical activity (HPLA) time and middle level physical activity (MPLA) time were both shorter in SSD children than in LSD children (both *p* < 0.05, Table 3), whereas daily walking and sedentary time were both longer in SSD children (both *p* < 0.05, Table 3).

**Table 3:** Sample characteristic across sleep duration groups among children aged 6–18 years in urban Guangzhou, China

	>9 hours (*n* = 1827)	7–9 hours (*n* = 4678)	<7 hours (*n* = 1414)	*P* value
Age (years)	9.2(2.3)	11.1(3.2)	14.6(2.6)	<0.001
Height(cm)	135.71(0.40)	145.34(0.27)	158.93(0.39)	<0.001
Weight(kg)	31.60(0.27)	39.47(0.23)	51.01(0.36)	<0.001
BMI(kg/m^2^)	16.6(0.09)	18.0(0.07)	19.9(0.13)	<0.001
Dietary intake(servings/day)				
Fruit	1.35(0.029)	1.20(0.015)	1.17(0.031)	<0.001
Vegetable	1.93(0.038)	1.91(0.023)	1.89(0.043)	0.765
Meat	1.53(0.032)	1.58(0.021)	1.70(0.039)	0.008
Sugar beverage	0.23(0.012)	0.34(0.009)	0.53(0.025)	<0.001
Physical activity (PA, hours/day)				
HLPA	0.46(0.014)	0.41(0.009)	0.39(0.015)	0.003
MLPA	0.47(0.016)	0.37(0.008)	0.36(0.015)	<0.001
Walking	0.84(0.028)	0.84(0.016)	0.93(0.029)	0.021
Sedentary	4.09(0.085)	4.53(0.054)	4.44(0.109)	<0.001
Obesity (%)	8.3	9.0	5.9	0.001
Age 6–12 years				
Boys	10.1	13.8	12.2	0.344
Girls	5.5	7.1	10.2	0.113
In total	8.8	10.1	10.5	0.211
Age 13–18 years				
Boys	4.7	9.1	5.3	0.016
Girls	3.8	4.7	5.1	0.856
In total	4.3	6.9	5.1	0.074

*BMI* body mass index, *PA* physical activity, *HLPA* high level physical activity, *MLPA* middle level activity

*P*-values are from comparison across groups of sleep duration using one-way ANOVA (continuous variables), or chi-square test (categorical variables)

Continuous variables are displayed as mean ± standard error

In total, the prevalence of obesity was age- and gender-dependent (see Table 3) and significantly different among sleep durations (*p* = 0.001, Table 3). For older boys, the prevalence of obesity was highest in MSD children, (9.1 %, *p* = 0.016, Table 3). The prevalence of obesity varied in other age and gender groups, but it did not reach the statistical significance (all *p* > 0.05, Table 3).

### Association between sleep duration and obesity

According to sleep duration, the odds ratios (ORs) for obesity are summarized in Table 4. Compared with LSD group, both SSD and MSD were significantly associated with obesity (ORs: 1.271 and 1.471, 95 % CI: 1.267–1.275 and 1.462–1.479, respectively, as shown in Table 4) after age and gender were adjusted, and multivariate adjusted analysis showed similar association (ORs: 1.581 and 1.328, 95 % CI: 1.572–1.590 and 1.324–1.332, SSD and MSD, respectively, as shown in Table 4).

**Table 4:** Odds ratio (OR) and 95 % confidence interval (CI) for obesity according to sleep duration among children aged 6–18 years in urban Guangzhou, China

	≥9 hours (*n* = 1827)	7–9 hours (*n* = 4678)	<7 hours (*n* = 1414)
Age, gender adjusted	1	1.271(1.267–1.275)	1.471(1.462–1.479)
Multivariate adjusted^a^	1	1.328(1.324–1.332)	1.581(1.572–1.590)
Genders			
Boys			
Age adjusted	1	1.207(1.201–1.214)	0.655(0.641–0.669)
Multivariate adjusted^a^	1	1.235(1.229–1.242)	0.700 (0.685–0.716)
Girls			
Age adjusted	1	1.313(1.307–1.318)	1.605(1.594–1.615)
Multivariate adjusted^a^	1	1.391(1.385–1.397)	1.725(1.714–1.736)
Age/gender groups			
Boys			
6–12 years			
Age adjusted	1	1.108(1.102–1.115)	0.624(0.570–0.683)
Multivariate adjusted^a^	1	1.121(1.114–1.127)	0.603(0.551–0.660)
13–18 years			
Age adjusted	1	2.126(2.099–2.153)	1.227(1.196–1.258)
Multivariate adjusted^a^	1	2.220(2.192–2.249)	1.331(1.297–1.365)
Girls			
6–12 years			
Age adjusted	1	1.310(1.304–1.316)	1.685(1.639–1.678)
Multivariate adjusted^a^	1	1.319(1.313–1.325)	1.748(1.728–1.768)
13–18 years			
Age adjusted	1	1.907(1.882–1.923)	2.400(2.366–2.434)
Multivariate adjusted^a^	1	2.247(2.217–2.277)	2.868(2.826–2.909)

^a^Adjusted for age, physical activity and inactivity, intake of fruit, vegetable, sugar beverage and meat

Stratified by gender, SSD was positively associated with obesity in girls but inversely associated with obesity in boys (age adjusted, ORs: 1.605 and 0.655, 95 % CI: 1.594–1.615 and 0.641–0.669, respectively, as shown in Table 3). MSD was positively associated with obesity in both genders (age adjusted, ORs: 1.207 and 1.313; 95 % CI: 1.201–1.214 and 1.307–1.318, boys and girls, respectively, as shown in Table 3). When multivariate adjusted (including age, dietary intake and physical activity/sedentary behaviour), the ORs of the groups were 1.235 (95 % CI: 1.229–1.242), 0.700 (95 % CI: 0.685–0.716); 1.391 (95 % CI: 1.385–1.397) and 1.725 (95 % CI: 1.714–1.736) (boys: MSD, SSD; girls: MSD, SSD, respectively, as shown in Table 3).

Stratified by age and gender, SSD was positively associated with obesity risk in older boys but inversely associated with obesity risk in younger boys (ORs: 1.331 and 0.603; 95 % CI: 1.297–1.365 and 0.551–0.660, respectively, as shown in Table 3). For girls, SSD was positively associated with obesity in both older and younger age groups (ORs: 2.868 and 1.748; 95 % CI: 2.826–2.909 and 1.728–1.768, respectively, as shown in Table 3). For boys with MSD, the association between sleep duration and obesity risk was stronger in the older group than in the younger group (ORs: 2.220 and 1.121; 95 % CI: 2.192–2.249 and 1.114–1.127, respectively, as shown in Table 3). MSD girls shared a higher obesity risk than their LSD counterparts (ORs: 2.247 and 1.319; 95 % CI: 2.217–2.277 and 1.313–1.325 for older and younger age groups, respectively, as shown in Table 3).

## Discussion

Childhood obesity has become an international epidemic as the risk of obesity-related morbidity increases in different countries. Considering the long-term potential complications of obesity, identifying the different etiologies of this disease is increasingly underscored [18]. Sleep duration is related to obesity [18–21] and influenced by numerous factors, such as gender, age, physical activity and dietary intake. However, information on this phenomenon is limited, particularly in the Chinese population. Using a cross-sectional survey on 8,760 children, we determined that the prevalence of childhood obesity was 8.4 % in the urban area of China, and was higher in boys than in girls. Moreover, sleep duration decreased as children grew. Most importantly, short sleep duration was related to the increased risk of obesity in girls and 13- to 18-year-old boys, whereas the decreased risk of obesity was found among boys aged 6–12 years. Generally, the results of this study suggest that the association between sleep duration and obesity is age- and gender-dependent, and pre-puberty boys can present an interesting population for further investigation.

Compared with the other well-known risk factors of obesity, such as parental obesity, TV viewing, socio-economic status and physical activity, short sleep duration was reported to be an independent risk factor of obesity [22]. The recommended sleep duration for a child is 9–11 h per night [23]. However, in the current study, more than half of the 6-year-old children could not get 9 h of sleep per night. Moreover, insufficient sleep became more serious with increasing age, and nearly 100 % of the children slept less than 9 h per night at age 18 (see Fig. 1). Epidemiological evidence shows that short sleep duration is associated with obesity, metabolic diseases and all-cause mortality [24]. Additionally, short sleep duration is linked with stress reaction [25]. As a stressor, long-lasting daily short sleep activates oxidative stress and systematic inflammation, which may finally induce the onset of chronic metabolic diseases [26]. Hence, proper sleep duration is critically important for the long-term health of children.

Although the association between short sleep duration and child obesity has been reported by cross-sectional studies, the underlying mechanism remains unclear. The imbalance of energy intake and expenditure is currently considered the most plausible reason for the increased risk of obesity of short duration sleepers [27]. Energy intake is regulated by ghrelin and leptin, which have opposing functions in appetite regulation [28]. Ghrelin stimulates hunger and increases food intake, whereas leptin suppresses appetite and improves energy expenditure [28]. Laboratory studies revealed that short sleep could increase ghrelin level and decrease leptin level in the human body, which may alter eating habits and eventually predispose weight gain and obesity in the future [28].

In this study, we observed the increased intake of meat and sugar beverages for children who had less than 7 h sleep (see Table 2). Although we did not test the ghrelin and leptin levels of participants, our results supported the laboratory findings of appetite hormone. Our findings are also consistent with epidemiological studies that reported the association between less sleep and more consumption of high-calorie food [29], such as sugar beverages [30]. Moreover, energy is believed to contribute to the association between short sleep and obesity. Insufficient sleep with increased fatigue and daytime sleepiness would eventually reduce the motivation to exercise [11, 31]. Additionally, sedentary behaviours, such as TV viewing and playing video games, proved to be important lifestyle factors of childhood obesity [32]. In our study, children with short sleep duration had less HLPA and MLPA, but more walking and sedentary behaviour time (see Table 2), which supported the aforementioned points. Several studies have explored the U-shape association between long or short sleep duration and obesity. However, when sleep duration is a continuous variable, the U-shape can be tested more easily. Considering that sleep duration is a categorical variable in our study, the U-shape association cannot be ruled out.

Numerous studies have reported that gender affects the relationship between short sleep and overweight/obesity [33]. For instance, several studies have suggested that the effect of sleep duration on obesity was only present in females with greater sleep debt [34]. A widespread belief is that females require less sleep hours than males [35]. We also determined that more girls had less than 7 h of sleep duration than boys (see Table 1). Given that the current study showed a significant obesity risk for girls with short sleep, less than 7 h of sleep duration is sufficiently short to predict the higher risk of obesity in females. Furthermore, our findings indicated that girls with less than 7 h of sleep duration had longer sedentary time, which explained the reason for higher risk of obesity.

Compared with girls, boys had a different pattern in the association between sleep and obesity. The results of this study revealed that pre-puberty boys (aged 6–12 years) with less than 7 h of sleep showed a decreased risk for obesity. From the perspective of energy intake and expenditure, sleeping reduces the basic metabolic rate and energy expenditure [36]. Considering that boys require more sleep hours than girls [35], less than 7 h of sleep for boys may be extremely short, which could induce more energy expenditure compared with girls. We also found that obesity prevalence among boys aged 13–18 years was higher in the 7–9 sleep-hour group than in the 7 sleep-hour group. Extra energy expenditure occurs in longer waking up time as well. Collings et al. [37] concluded that adiposity was inversely associated with sleep duration among adolescent boys, but not girls, which further confirmed the gender-specific effect. The preceding speculation indicates the worthiness of exploring the mechanism of short sleep effect on obesity from the perspective of energy intake and expenditure in the future.

In addition to gender, puberty seems an important factor in the effect of obesity. In the dynamic developmental period of physical and sexual development, adolescents require adequate time of sleep. However, no difference was found between the period of pre-puberty and puberty in females in this study. For boys, puberty induces a dramatic change, and less than 7 h of sleep could turn from a protective factor to become a risk of obesity. However, the role of puberty is unclear in the effect of sleep on obesity among boys. We speculate that rapid endocrine and metabolic change during puberty might be one reason. Additionally, the requirement of sleep time in girls is relatively shorter compared with boys; thus, the influence of short sleep duration on boys is more obvious [35]. The inner mechanism that puberty changes the pattern of sleep and obesity is warranted.

Other aspects may affect the association between obesity and sleep duration. Later bedtime is regarded as a risk factor of increased BMI [38], even if sleep duration remains the same. Sleep quality should be considered as well because poor sleep quality can be a risk factor of obesity [39]. Sleep apnea can be an independent risk factor of obesity [40]. However, obesity-related sleep apnea could result in sudden awakening at night and decrease sleep duration. Although Colings et al. [15] previously reported the lack of association between adiposity and changes in sleep duration from mid- to late adolescence, the reverse causality of the association cannot be ruled out. As a cross-sectional study, we did not aim to explore the causality, but our findings may help enhance the understanding on the association itself, such as potential gender-specific effect.

The population in our study consists of children in the urban area of Guangzhou. Guangzhou is the third largest city in China, which is located along the south coast. With regard to economic development, cities along the east and south coast are more developed relative to inland and west cities of China; consequently, the average income is higher in Guangzhou. Additionally, cities in the south differ from those in north China in terms of lifestyle (i.e. diet preference), which may potentially affect the external validity. In conclusion, our study can be representative when referring to children in urban cities in southern China.

Several limitations are found in this study. Given that the study was conducted in a cross-sectional manner, the causal pathways underlying the observed relationships could hardly be detected. The questionnaire has not been statistically validated nor tested for reliability; thus, potential bias cannot be excluded. Sleep duration was reported by the parents of children under nine years old, which sometimes could be an ideal sleep duration that the parents think their children had other than the latter’s actual sleep duration [41]. Children older than nine years personally reported the information, which may have self-reported bias. In addition, nearly 25 % of participants either failed to complete the anthropometric procedure or refused to answer the questionnaire. Obese children felt might feel embarrassed to be weighed, which may induce a relatively low prevalence of obesity. Thus, the excluded subjects may have a higher rate of obesity. An objective technique is considerably useful in conducting sleep research and generally deemed to be the optimal manner. Although diverse lifestyle behaviours were adjusted, residual confounding remained in the models because of methodological imperfection, which may compromise the results.

## Conclusion

Overall, short sleep duration is associated with obesity among children. Short sleep duration increases the obesity risk for girls and 13- to 18-year-old boys, but decreases the obesity risk for boys aged 6–12 years in the urban area of China. Data suggest that age and gender should be regarded as specific characteristics for the effects of short sleep on obesity. Further research should clarify the age- and gender-specific effects on the relationship between sleep and obesity, and explore the underlying mechanisms.

## References

[1] Mindell JA, Owens JA, Carskadon MA (1999). Developmental features of sleep. Child Adolesc Psychiatr Clin N Am.

[2] Van Cauter E, Knutson KL (2008). Sleep and the epidemic of obesity in children and adults. Eur J Endocrinol.

[3] Kripke DF, Simons RN, Garfinkel L, Hammond EC (1979). Short and long sleep and sleeping pills. Is increased mortality associated?. Arch Gen Psychiatry.

[4] Organization G: Sleep in america: 1995 gallup poll., Princeton, 1995.

[5] Drobnich D (2005). A National Sleep Foundation’s conference summary: The National Summit to Prevent Drowsy Driving and a new call to action. Ind Health.

[6] Shi Z, Taylor AW, Gill TK, Tuckerman J, Adams R, Martin J (2010). Short sleep duration and obesity among Australian children. BMC Public Health.

[7] Li S, Zhu S, Jin X, Yan C, Wu S, Jiang F (2010). Risk factors associated with short sleep duration among Chinese school-aged children. Sleep Med.

[8] Bornhorst C, Hense S, Ahrens W, Hebestreit A, Reisch L, Barba G (2012). From sleep duration to childhood obesity--what are the pathways?. Eur J Pediatr.

[9] Stamatakis KA, Brownson RC (2008). Sleep duration and obesity-related risk factors in the rural Midwest. Prev Med.

[10] Martinez SM, Tschann JM, Greenspan LC, Deardorff J, Penilla C, Flores E (2014). Is it time for bed? Short sleep duration increases risk of obesity in Mexican American children. Sleep Med.

[11] Chaput JP, Lambert M, Gray-Donald K, McGrath JJ, Tremblay MS, O’Loughlin J (2011). Short sleep duration is independently associated with overweight and obesity in Quebec children. Can J Public Health.

[12] Kong AP, Wing YK, Choi KC, Li AM, Ko GT, Ma RC (2011). Associations of sleep duration with obesity and serum lipid profile in children and adolescents. Sleep Med.

[13] Hitze B, Bosy-Westphal A, Bielfeldt F, Settler U, Plachta-Danielzik S, Pfeuffer M (2009). Determinants and impact of sleep duration in children and adolescents: Data of the Kiel Obesity Prevention Study. Eur J Clin Nutr.

[14] Fatima Y, Doi SA, Mamun AA (2015). Longitudinal impact of sleep on overweight and obesity in children and adolescents: A systematic review and bias-adjusted meta-analysis. Obes Rev.

[15] Collings PJ, Wijndaele K, Corder K, Westgate K, Ridgway CL, Sharp SJ (2015). Magnitude and determinants of change in objectively-measured physical activity, sedentary time and sleep duration from ages 15 to 17.5y in UK adolescents: The ROOTS study. Int J Behav Nutr Phys Act.

[16] Nugent R, Althouse A, Yaqub Y, Nugent K, Raj R (2014). Modeling the relation between obesity and sleep parameters in children referred for dietary weight reduction intervention. South Med J.

[17] Li H, Zong XN, Ji CY, Mi J (2010). Body mass index cut-offs for overweight and obesity in Chinese children and adolescents aged 2–18 years (in Chinese). Zhonghua Liu Xing Bing Xue Za Zhi.

[18] Kelly-Pieper K, Lamm C, Fennoy I (2011). Sleep and obesity in children: A clinical perspective. Minerva Pediatr.

[19] Kjeldsen JS, Hjorth MF, Andersen R, Michaelsen KF, Tetens I, Astrup A (2014). Short sleep duration and large variability in sleep duration are independently associated with dietary risk factors for obesity in Danish school children. Int J Obes (Lond).

[20] Magee C, Caputi P, Iverson D (2014). Lack of sleep could increase obesity in children and too much television could be partly to blame. Acta Paediatr.

[21] Perrier B (2014). [in children, obesity and lack of sleep are linked]. Rev Med Suisse.

[22] Chaput JP, Brunet M, Tremblay A (2006). Relationship between short sleeping hours and childhood overweight/obesity: Results from the ‘Quebec en Forme’ Project. Int J Obes (Lond).

[23] Skelton JA (2013). Family intervention focused on effective parenting is associated with decreased child obesity prevalence 3–5 years later. Evid Based Med.

[24] Cappuccio FP, D’Elia L, Strazzullo P, Miller MA (2010). Sleep duration and all-cause mortality: A systematic review and meta-analysis of prospective studies. Sleep.

[25] Vgontzas AN, Bixler EO (2008). Short sleep and obesity: Are poor sleep, chronic stress, and unhealthy behaviors the link?. Sleep.

[26] Gishti O, Gaillard R, Durmus B, Abrahamse M, van der Beek EM, Hofman A, et al. Body mass index, total and abdominal fat distribution and cardiovascular risk factors in school-age children. Pediatr Res. 2015;10–1038. 10.1038/pr.2015.29 25665058

[27] Reilly JJ, Ness AR, Sherriff A (2007). Epidemiologic and physiologic approaches to understanding the etiology of pediatric obesity: Finding the needle in the haystack. Pediatr Res.

[28] Prinz P (2004). Sleep, appetite, and obesity--what is the link?. PLos Med.

[29] McDonald L, Wardle J, Llewellyn CH, Fisher A. Nighttime sleep duration and hedonic eating in childhood. Int J Obes (Lond). 2015; doi:10.1038/ijo.2015.132 10.1038/ijo.2015.132 PMC4597336 26189601

[30] Weiss A, Xu F, Storfer-Isser A, Thomas A, Ievers-Landis CE, Redline S (2010). The association of sleep duration with adolescents’ fat and carbohydrate consumption. Sleep.

[31] Taheri S (2006). The link between short sleep duration and obesity: We should recommend more sleep to prevent obesity. Arch Dis Child.

[32] Baranowski T, Mendlein J, Resnicow K, Frank E, Cullen KW, Baranowski J (2000). Physical activity and nutrition in children and youth: An overview of obesity prevention. Prev Med.

[33] Guidolin M, Gradisar M (2012). Is shortened sleep duration a risk factor for overweight and obesity during adolescence? A review of the empirical literature. Sleep Med.

[34] Eisenmann J, Ekkekakis P, Holmes M (2006). Sleep duration and overweight among Australian children and adolescents. Acta Paediatr.

[35] Keyes KM, Maslowsky J, Hamilton A, Schulenberg J (2015). The great sleep recession: Changes in sleep duration among US adolescents, 1991–2012. Pediatrics.

[36] FRASER R, NORDIN BE (1955). The basal metabolic rate during sleep. Lancet.

[37] Collings PJ, Wijndaele K, Corder K, Westgate K, Ridgway CL, Sharp SJ (2015). Prospective associations between sedentary time, sleep duration and adiposity in adolescents. Sleep Med.

[38] Asarnow LD, McGlinchey E, Harvey AG. Evidence for a possible link between bedtime and change in body mass index. Sleep 2015 10.5665/sleep.5038 PMC4576325 26194568

[39] Sun W, Yuan J, Yu Y, Wang Z, Shankar N, Ali G et al. Poor sleep quality associated with obesity in men. Sleep Breath 2015; doi:10.1007/s11325-015-1193-z 10.1007/s11325-015-1193-z 25957618

[40] Bonuck K, Chervin RD, Howe LD (2015). Sleep-disordered breathing, sleep duration, and childhood overweight: A longitudinal cohort study. J Pediatr.

[41] Short MA, Gradisar M, Lack LC, Wright HR, Chatburn A (2013). Estimating adolescent sleep patterns: Parent reports versus adolescent self-report surveys, sleep diaries, and actigraphy. Nat Sci Sleep.

